# *Prevotella histicola* Protects From Arthritis by Expansion of *Allobaculum* and Augmenting Butyrate Production in Humanized Mice

**DOI:** 10.3389/fimmu.2021.609644

**Published:** 2021-05-04

**Authors:** Baskar Balakrishnan, David Luckey, Rahul Bodhke, Jun Chen, Eric Marietta, Patricio Jeraldo, Joseph Murray, Veena Taneja

**Affiliations:** ^1^ Department of Immunology, Mayo Clinic, Rochester, MN, United States; ^2^ National Center for Microbial Resource, National Center for Cell Science, Pune, India; ^3^ Department of Biomedical Statistics and Informatics, Mayo Clinic, Rochester, MN, United States; ^4^ Department of Medicine, Division of Gastroenterology and Hepatology, Mayo Clinic, Rochester, MN, United States; ^5^ Department of Surgery, Division of Surgical Research, Mayo Clinic College of Medicine, Rochester, MN, United States

**Keywords:** *Prevotella histicola*, bio-therapeutics, rheumatoid arthritis, gut-microbiome, gut modulation, *Allobaculum*

## Abstract

Bacterial therapeutics are the emergent alternatives in treating autoimmune diseases such as Rheumatoid Arthritis [RA]. *P. histicola* MCI 001 is one such therapeutic bacterium that has been proven to treat autoimmune diseases such as RA and multiple sclerosis [MS] in animal models. The present study characterized *P. histicola* MCI 001 isolated from a human duodenal biopsy, and evaluated its impact on the gut microbial and metabolic profile in a longitudinal study using the collagen-induced arthritis model in HLA-DQ8.AEo transgenic mice. *P. histicola* MCI 001 though closely related to the type strain of *P. histicola*, DSM 19854, differed in utilizing glycerol. In culture, *P. histicola* MCI 001 produced vitamins such as biotin and folate, and was involved in digesting complex carbohydrates and production of acetate. Colonization study showed that duodenum was the predominant niche for the gavaged MCI 001. A longitudinal follow-up of gut microbial profile in arthritic mice treated with MCI 001 suggested that dysbiosis caused due to arthritis was partially restored to the profile of naïve mice after treatment. A taxon-level analysis suggested an expansion of intestinal genus *Allobaculum* in MCI001 treated arthritic mice. Eubiosis achieved post treatment with *P. histicola* MCI 001 was also reflected in the increased production of short-chain fatty acids [SCFAs]. Present study suggests that the treatment with *P. histicola* MCI 001 leads to an expansion of *Allobaculum* by increasing the availability of simple carbohydrates and acetate. Restoration of microbial profile and metabolites like butyrate induce immune and gut homeostasis.

## Introduction

A diverse adaptive behavior of the genus *Prevotella* leads to high level of species and strain diversity. Characters of the strains are decided by the habitat in which they live and adapt; some strains of *Prevotella* are regarded as part of an oral community and cannot colonize the gut, while other *Prevotella* do colonize the gut (1–3). Oral bacteria traverse through various parts of the intestine along with food and saliva mixture. However, only a few bacteria survive and can colonize the gut. *Prevotella* is one of the most abundant bacterial populations in the oral and gut microbiome. Hence discovering and understanding the novel strains of the genus *Prevotella* is a key to discovering some of the uncharacterized aspects of the human microbiome.

There are four genera present in family *Prevotellaceae* with 54 recorded species, among those, 50 species belong to the genus *Prevotella*. There are two groups of *Prevotella* based on their biochemical characters. The first is the *P. melaninogenica* group that ferments lactose and does not produce indole; members of this group are regarded as commensal microbiota. The second is the *P. intermedia* group; members of this group produce indole and are dominant species in periodontal diseases (1, 4). *P. histicola* has received much attention for its various habitual adaptations and its biomedical importance. *P. histicola* was initially isolated from human oral mucosal tissue associated with oral squamous cell carcinoma by Hooper et al. (5) and then taxonomically classified by Downs et al. (6). Based on the site of isolation, *P. histicola* was initially considered to be an oral commensal bacterium.

Recently, a role of gut microbiome has gained increased attention due to its impact on immune system (7–10). The function and diversity of the microbial population in gut plays a vital role in many biological processes of the host; alterations in microbial composition lead to an imbalance in the host’s metabolic activity. Dysbiosis in the gut microbiome is associated with various autoimmune diseases (8, 11, 12). The newfound importance of the gut microbiota has raised the question of manipulating the gut microbial and metabolic composition for treating disease conditions (8, 13, 14). Modulation of gut microbiota can be achieved through a variety of ways, such as prebiotic diet, antimicrobial interventions, fecal microbiota transplantation, and probiotics (8, 15, 16). Among the gut modulation options, use of novel therapeutic bacteria increases the potential of treating diseases by targeted microbial restoration in the gut, whereas other options are mostly untargeted (8).

Generally probiotics have a broad spectrum of activities such as digestion of complex nutrients, enhancing the gut immune homeostasis by producing secondary metabolites such as SCFAs and reducing pathogenic bacteria (9, 17, 18). SCFAs are essential metabolites in the gut that regulates the immune system by directly interacting with immune cells present in the gut ecosystem (19). SCFAs such as butyrate can activate GPR43-expressing T regulatory cells in keeping gut immune homeostasis (19, 20). Hence SCFA producing gut microbial candidates are widely tested for their potential in using therapeutic probiotics (8, 9). The prevalent over-the-counter probiotics are nonspecific and improve gut health for a short period (8). Recent studies on bacterial therapeutic approaches with anaerobic gut bacteria have opened the door to novel non-vaccinal biotherapeutic agents (21–24). Hence it is necessary to find indigenous anaerobic bacterial candidates as biotherapeutic agents for treating autoimmune and metabolic disorders (12). The use of selective bacterial members has shown improved immune response with better outcomes in mouse models of various diseases (8, 12, 25, 26). The relationship between the presence of the genus *Prevotella* in the human gut and autoimmune diseases has been controversial with one species reported as pathogenic while another species was observed to be beneficial (11, 27–29).

Recently, we isolated a novel strain of *P. histicola, (*referred to as MCI001), from a human duodenal biopsy; it was the first report of its presence in the human gut (26). Using mouse models of RA and MS, we have shown that the strain MCI001 can regulate host’s immune system and prevent autoimmunity in mice (30, 31). Here we have characterized this novel strain taxonomically using phenotypic and phylogenetic classification. Furthermore, gut colonization characteristics of the strain were assessed for identifying it as a potential bacterial therapeutics using a humanized mouse model of arthritis. We also evaluated modulation of gut microbiota and metabolites by MCI001 and implications for its use in treating RA.

## Materials and Methods

### Characterization of *P. histicola* MCI001

Standard methods were followed for the physiological and biochemical tests (32, 33). Classification and identification of the strain was resolved based on *Bergey’s Manual of Systematics of Archaea and Bacteria* (34). Vitamin biosynthesis was analyzed using microbiological assay methods (35). For phenotypic characterization of MCI001, commercially available API kits were used [API 50 CH, API 20E, API ID 32 and API ZYM; bioMe´rieux, Germany]. In all experiments, type strain *P. histicola* DSM 19854 and *P. melaninogenica* ATCC 25845 were used as controls. Colony morphology was observed by culturing microbes on tryptic soy agar [TSA] and tryptic soy blood agar plates. For broth culture, tryptic soy broth [TSB] was used. The culture conditions were kept at 37°C for 24 – 96 hours, with pH of 7.2 ± 0.2. Susceptibility to antimicrobial agents was tested using commercially prepared antimicrobial discs [BD Life Sciences, USA]. For electron microscopy, bacteria cultured for 36 hrs. in TSB were collected through centrifugation followed by washing with PBS and suspended in Trump’s fixative for slide preparation. Transmission Electron Microscopy [TEM] was done using a Philips CEM 300 electron microscope at Electron Microscopy Facility at Mayo Clinic, Rochester, USA.

### Tolerance to Bile Salts and Low pH

The resistance of MCI001 to different concentrations of bile salts and low pH in culture condition was analyzed. For resistance to low pH, Phosphate Buffer Saline (PBS) was adjusted to variable pH; 2.0, 2.5, 3.0, 3.5 and 4.5. Similarly, PBS was prepared with different concentrations of bile salts [0.25, 0.5, 0.75 and 1.0(w/v)]. Prepared PBS solution was inoculated with 0.5 mL MCI001 (10^9^ CFU ml^-1^) cultured for over 36 hours, and incubated for 3 hrs. in anaerobic condition as mentioned above. Cultures were then subjected to serial dilutions followed by plating on blood agar plates and incubating for 48 – 72 hrs. to enumerate the Colony Forming Unit (CFU) for the survival rate of MCI001 in treated condition. Triplicates were maintained for each experiment.

### Tolerance to Gastric Juice

To evaluate the tolerance of MCI001 in gastric juice, a simulated gastric juice was used (Sigma Aldrich, USA). Modified TSB was used in the preparation of simulated gastric juice, where the pH was adjusted to pH 2 using hydrochloride (HCl). Sterile TSB was supplemented with 0.25% bile salt, pepsin (15 µg ml^-1^) and Lysozyme (100 µg ml^-1^). Prepared gastric juice was inoculated with 0.5 mL of MCI001 culture (10^9^ CFU ml^-1^) (cultured for 36 hrs.) and incubated for 3 hrs. in anaerobic condition and CFU analysis was carried out using serial dilution method.

### 16SrRNA Gene Sequencing and Phylogeny

For 16S rRNA gene sequencing, genomic DNA of the pure culture of MCI001 was isolated [PowerDNA isolation, QIAGEN, USA] and subjected to PCR amplification using a universal 16S rDNA primer set (**Table 1**). PCR amplification was carried out using KAPA HiFi Hotstart PCR ready mix [KAPA Biosciences, USA]. After amplification, the product was sequenced [Sangers sequencing] using the same primers by the Genomics facility at Mayo Clinic, Rochester, USA. BioEdit program was used to assemble the sequences (39). The closest relatives of the strain were identified using BLAST analysis. Selected sequences were aligned using CLUSTAL W program, and the phylogenetic relationship was analyzed, and the tree was constructed using MEGA7 program with neighbor-joining method (40).

**Table 1:** Primers used in this study.

Primers	Designation	Sequence [5′ to 3′]	Position [by *E. coli* Numbering]	Amplicon size [bp]	Reference
Universal 16S	27F 1492R	AGAGTTTGATCCTGGCTCAG GGTTACCTTGTTACGACTT	27 – 1492	~1400	(36)
Universal 16S qPCR	V4F_517 V4R_805	AGGCAGCAGTGGGGAAT GCCAGCAGCCGCGGTAA	517 – 805	~289	(37)
*Prevotella* qPCR	g-Prevo-F g-Prevo-R	CACRGTAAACGATGGATGCC GGTCGGGTTGCAGACC	– –	527–529	(38)
*P. histicola* qPCR	PhisF Phis1R	TCACTGACGGCATCAGATGTG CAATCACACGTGACTGACT	162-183 450-433	289	(4)

### Induction of Collagen-Induced Arthritis and Treatment with *P. histicola* MCI001

Transgenic mice lacking major histocompatibility complex class II [MHC II] genes [AEo] and expressing the human HLA-DQB1*0302/DQA1*0301 gene [DQ8.AEo] on the B6/129 background were generated as described previously (41). Mice aged 8-12 weeks used in the study were bred and maintained in a pathogen-free colony by the Department of Comparative Medicine at Mayo Clinic, Rochester, MN. All animal experiments used in the study were approved by the Institutional Animal Care and Use Committee [IACUC protocol # A00002667-17].

To determine the effect of MCI001 treatment over a period, longitudinal study was conducted using DQ8 mice [n=15] which helped in avoiding cage effects as confounding factors. Induction and evaluation of collagen-induced arthritis [CIA] were carried out by immunization with type II collagen [CII] and follow-up of arthritis progression as described previously (26). Fecal samples were collected prior to the induction of arthritis and then at varying times. Mice were divided into 3 groups, Gp.1- Naïve control [n=5], un-manipulated, without any treatments and immunizations, Gp.2- disease control [n=5], CII-immunized and no bacterial treatment and Gp.3- mice immunized with CII and subjected to bacterial treatment. Mice were immunized with collagen [n=5] and treated with MCI001 [100µL of 10^9^ CFU ml^-1^] one week after immunization on alternate days for 5 weeks. Fecal samples were collected at various time points for each mouse separately and stored in -80°C [day 0, one week post-immunization, after 2 weeks of bacterial treatment and at the end of the experiment]. Mice were sacrificed 6 weeks post-immunization and various gut sections duodenum, jejunum, ileum, caecum, and colon were collected for microbiome analysis (26). Intestinal sections were gently washed with PBS to remove debris and the mucus layer was scrapped out. For cecum, whole cecal content along with mucus layer was used. From each intestinal section, 100 mg of the collected sample was used for DNA isolation procedure.

### 16S Sequencing for Microbiome Analysis

DNA isolation from gut sections and fecal samples was carried out using QIAamp PowerFecal DNA Isolation Kit [QIAGEN, USA]. To prepare libraries, a polymerase chain reaction [PCR] was performed using a universal forward primer targeting 357F [**5′**AATGATACGGCGACCACCGAGATCTACACTATGGTAATTGTCCTACGGGAGGCAGCAG**3′**] and barcoded reverse primer targeting 926R [**5′**CAAGCAGAAGACGGCATACGAGATNNNNNNNNNNNNAGTCAGTCAGCCCCGTCAATTCMTTTRAGT**3′**] (42) using Kapa HiFi Hotstart Ready PCR Mix [Kapa Biosystems]. The amplified product was checked for quality by agarose gel electrophoresis, and the PCR product was subjected to purification using Agencourt AMPure XP beads [Beckman Coulter, USA] and quantified using NanoDrop [Thermo Scientific, USA]. An equal amount of DNA from each amplified sample was pooled for library preparation, concentrated by vacuum drying method using SpeedVac concentrator [SAVANT] and then resuspended in nuclease-free molecular biology grade sterile water [Sigma Aldrich, USA]. Final purification of the library was carried out using AMPure XP beads and final DNA concentration calculated using Qubit fluorimeter [Invitrogen, USA]. 16S rRNA gene sequencing was done at the Mayo Genomics Facility using the MiSeq Reagent Kit v2 [500 cycles; Illumina Inc.], generating 20 M 2x250 reads. Sequence reads were clustered into operational taxonomic units [OTUs] by the bioinformatics pipeline *hybrid-denovo* [default parameter setting], which was built upon the IM-TORNADO pipeline to handle a mixture of good quality paired-end and single-end reads (11, 42, 43). The outputs of the pipeline [OTU abundance, phylognetic tree and OTU taxonomic assignments] were subjected to downstream community analysis.

### Microbial Community Analysis

A total of 7,746,101 reads with the taxonomic assignment averaging 127,663 ± 41396 reads per sample were obtained. The alpha and beta microbial diversity was analyzed with the R-packages phyloseq [v1.22.3] (44), microbiomeseq [https://github.com/umerijaz/microbiomeSeq] and microbiome[v1.0.2] [https://github.com/microbiome/microbiome]. Alpha diversity measures of three groups were compared using ANOVA. Bray-Curtis distance analysis was used for analysis of similarities [ANOSIM, package v 2.4-4] to test for similarities in microbial communities and generation of principal coordinate analysis [PCoA] plots (45). Differential abundance analysis of taxa in fecal samples between sample groups was performed by Wilcoxon rank sum test [Mann-Whitney test]. Due to a small sample size, multiple testing corrections were not performed. Taxa with nominal p-values less than 0.05 were subject to further follow-up. All analysis was done using R packages and data was visualized using ggplot2 [v 2.2.1] in R (46).

### Quantitative PCR

To assess the colonization character of MCI001 in gut, DNA isolated from the gut samples were subjected to qPCR quantification as described previously (4). Total bacterial population was quantified using universal qPCR 16S rRNA gene primer set. For total *Prevotella* and *P. histicola* quantifications, published specific primers were used (4). List of primers used in this study is given in **Table 1**.

### SCFAs

SCFAs in the fecal samples were quantified by liquid chromatography Mass spectrophotometer [LC-MS] by the Mayo Core facility. The obtained results were statistically analyzed with one-way ANOVA and plots generated using IBM SPSS statistics version 22.0.

## Results

### Description of *P. histicola* MCI001

MCI001 is an obligate anaerobe, non-motile and Gram-negative bacillus with the size of ~0.2-2.0 um in diameter. Colonies are ~1 -2 mm in diameter, entire convex circular, creamy and opaque on TSA. The β-hemolytic bacterium produces bull’s eye colonies with red to black pigmentation in the center of the colony after 2 to 4 days of incubation on blood agar plates. There is a moderate to high turbid growth in TSB after 24 to 48 hrs. of incubation, subsequently cells settle at the bottom of the tube suggesting a stationary phase of growth in 48 hrs. It is a saccharolytic strain and ferments fructose, galactose, glucose, glycogen, Inulin, lactose, maltose, mannose, raffinose, saccharose, sucrose, and turanose (**Table S1**). It does not ferment arabinose, arabinose, cellobiose, dulcitol, fucose, gentiobiose, inositol, lyxose, mannitol, melezitose, melibiose, rhamnose, ribose, salicin, sorbitol, sorbose, tagatose, trehalose, xylitol, and xylose. MCI001 produces acetic acid as the primary metabolic end product as well as succinic acid along with trace amount of isovaleric acid and lactic acid. MCI001 hydrolyzed gelatin but not aesculin, arginine, and urea and did not produce catalase, indole and nitrate reductase. The Rapid ID 32A profile is 4707 4502 22.

**Figure 1:**
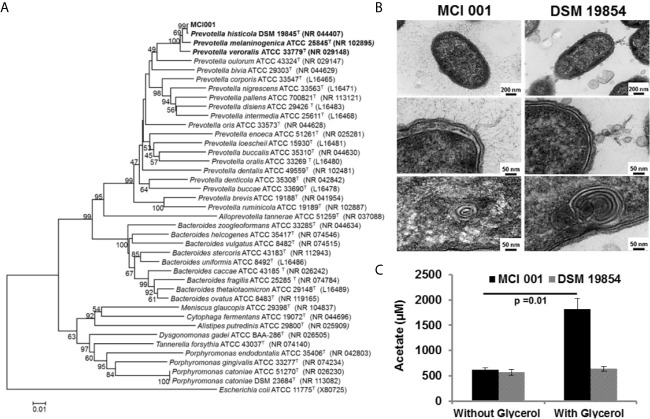
**(A)** Neighbor-Joining phylogenetic tree of *P. histicola* MCI001 constructed with closest relatives. The bootstrap consensus tree inferred from 1000 replicates was taken to represent the evolutionary history of the taxa analyzed. **(B)** Transmission electron microscope structures of *P. histicola* MCI 001 compared type strain of *P. histicola* DSM 19854. **(C)** Production of acetate in *in-vitro* cultures of *P. histicola* MCI 001 and the type strain of *P. histicola* DSM 19854 with or without glycerol in the culture medium.

The phylogenic analysis showed the MCI001 to be closest to type strain of *P. histicola* followed by, *P. jejuni, P. melaninogenica* and *P. veroralis* (**Figure 1A**). TEM structures from the ultra-thin sections of MCI001 showed similarities to the reported *Prevotella* members (47, 48) (**Figure 1B**). MCI001 TEM structure was a match with the tested type strain of *P. histicola* [DSM 19845]. Fundamental structures observed in TEM include space between the cell wall and cell membrane which holds gas vesicles and thread-like structures is coming out from the cell that carry loosely attached gas vesicles. TEM also showed fimbria structures all over the cell surface. Critical phenotypic characters of MCI001 that differ from the phylogenetically closest bacteria are listed in **Table 2**. All other phenotypic results are given in supplementary information (**Table S1**).

**Table 2:** Critical phenotypic characters of MCI001, distinguishes from closest relatives.

Test	MCI001	1	2	3	4
Pigment	+	+	+	+	–
Arabinose	–	–	–	–	–
Cellobiose	–	–	–	–	+
Lactose	+	+	+	+	+
Mannose	+	+	+	+	+
Raffinose	+	+	+	+	+
Salicin	–	–	–	–	–
Sucrose	+	+	+	+	+
Indole	–	–	–	–	–
Aesculin	–	–	–	–	+
Gelatin	+	+	+	+	–
Glycerol	+	–	–		NA
His-Ary	–	–	W	W	NA
Leu-Ary	–	–	–	+	NA
α-Gal	+	+	+	–	+
Sialidase	–	–	+	+	NA

****1, P. histicola DSM 19845^T^; ****2, P. melaninogenica ATCC 25845^T^; ****3, P. jejuni DSM 26989^T^; ****4, P. veroralis ATCC 33779^T^; Pigment, on blood agr; +, Positive; -, Negative; NA, not analyzed, W, weakly positive; His-Ary, Histidine Arylamidase; Leu-Ary, Leucine Arylamidase; α-Gal, α-Galactosidase.

MCI001 produces vitamins such as folate and biotin. This strain also metabolizes glycerol to produce high amounts of acetic acid, (**Figure 1C**). On the other hand, type strain DSM 19845 did not utilize glycerol and did not show increased acetate production (**Figure 1C**). MCI001 exhibits tolerance to bile salts [up to 0.5%], lower pH [pH 2] and gastric juice [up to 90 min], which are characteristics of a gut commensal colonizing small intestine (49).

### *P. histicola* MCI001 Treatment Partially Normalizes Gut Microbiota in DQ8 Mice

**Figure 2:**
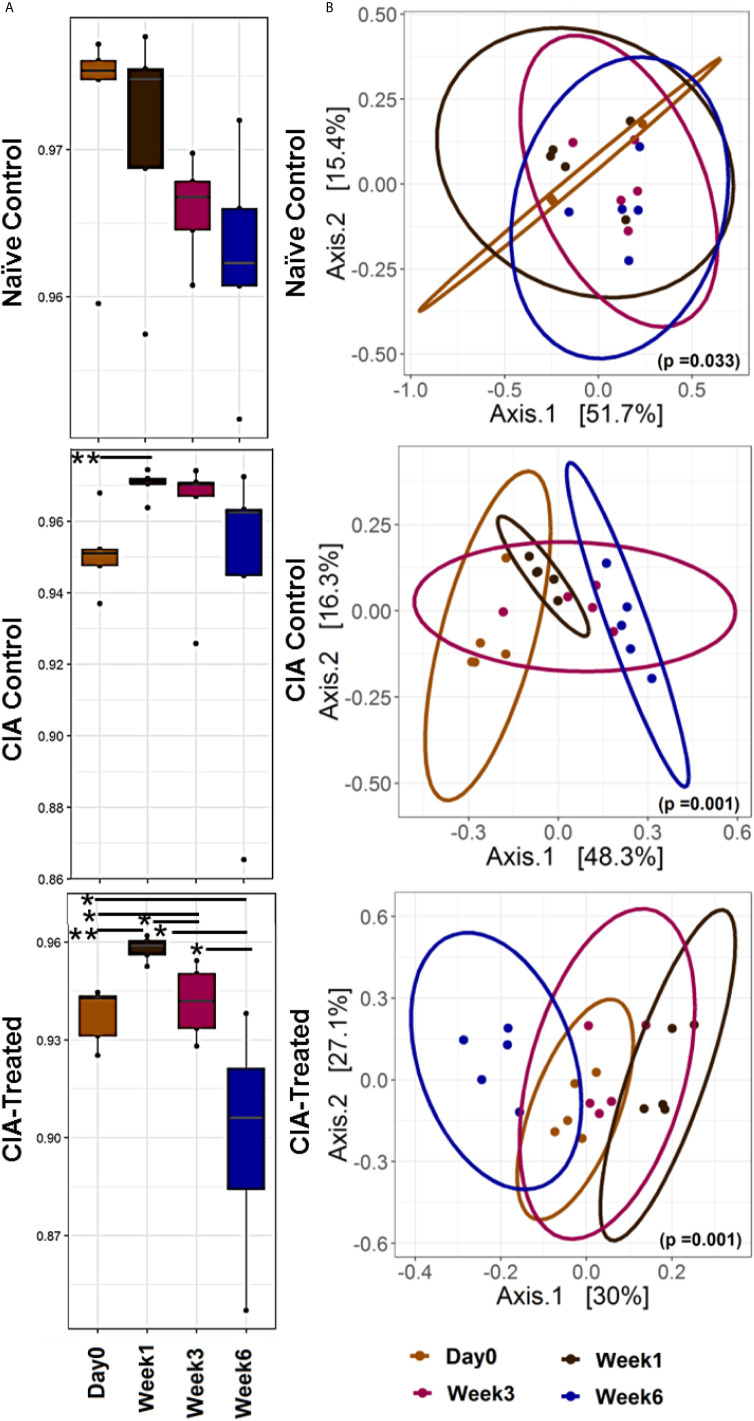
Treatment with MCI001 partially restores gut microbiota to naïve state. **(A)** A longitudinal analysis of alpha diversity (Simpson index) in fecal samples compared between various groups and time points. **(B)** Bray Curtis dissimilarity distance analysis illustrating the beta diversity of the fecal samples in various groups as indicated. N=5 for each group and time; statistical significance given as *P ≤ 0.05 and **P ≤ 0.01.

A longitudinal follow up for six weeks of fecal microbiota from naïve DQ8 mice showed a gradual decrease in alpha diversity using Simpson index (**Figure 2A**). An increase in genera *Allobaculum* and *Sutterella* with a decrease in *Coprococcus* and *Ruminococcus* in naïve mic*e* represent the healthy gut microbiome of DQ8 mice. As compared to naïve state, CII-immunization led to a microbial shift as indicated by apparent separation in alpha and beta diversities suggesting potential dysbiosis (**Figure 2**). Immunized mice showed a shift in beta diversity one week after CII-immunization. On the other hand, in CII-immunized mice treated with MCI001, the microbial profile was restored with similarities to naïve mice within 2 weeks of treatment suggesting that the dysbiosis caused by CII-immunization can be reversed by MCI001 treatment (**Figure 2B**).

**Figure 3:**
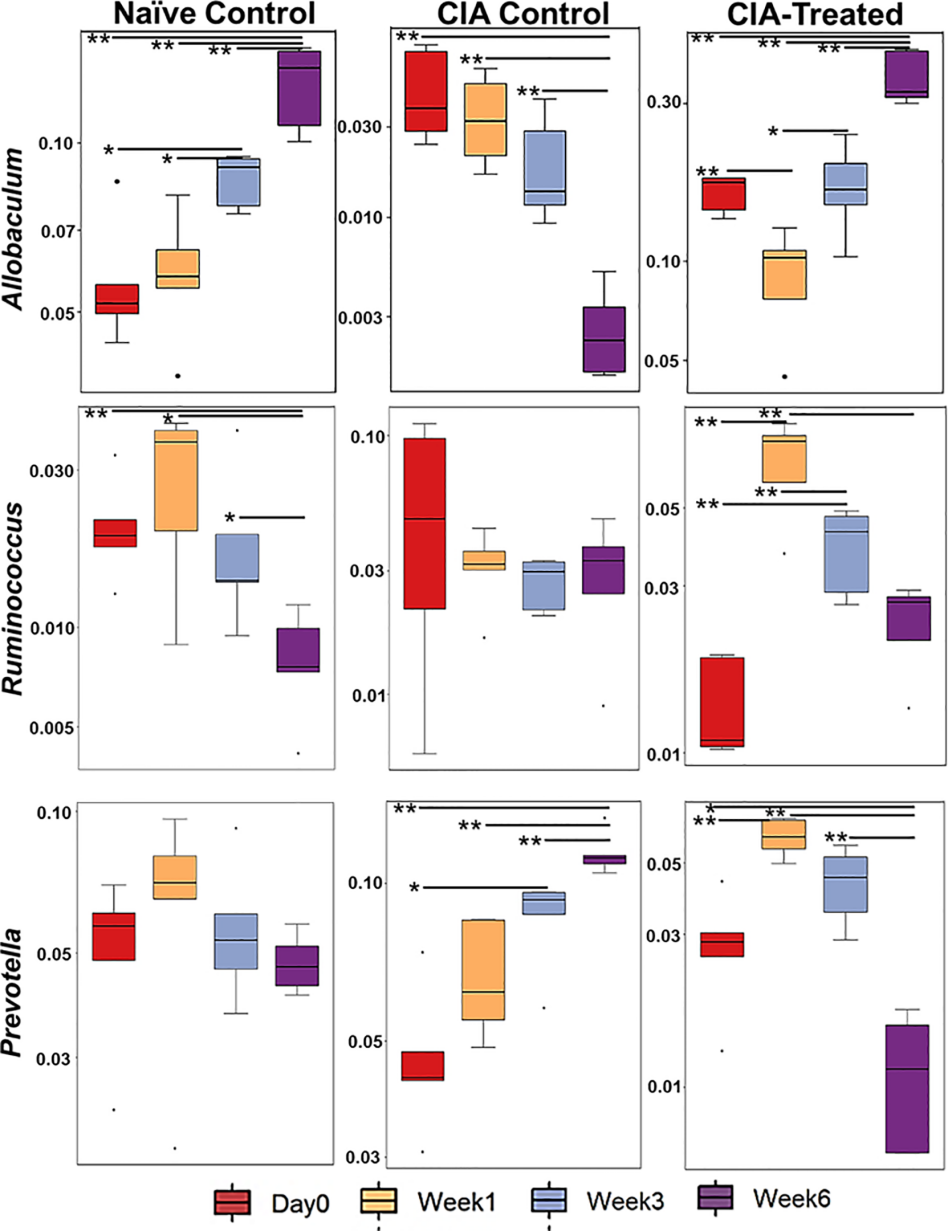
A comparison of the relative abundance of the most significant bacteria [*Allobaculum*, *Prevotella*, and *Ruminococcus*] at various time points. N=5 for each group and time; statistical significance at *P ≤ 0.05 and **P ≤ 0.01.

**Figure 4:**
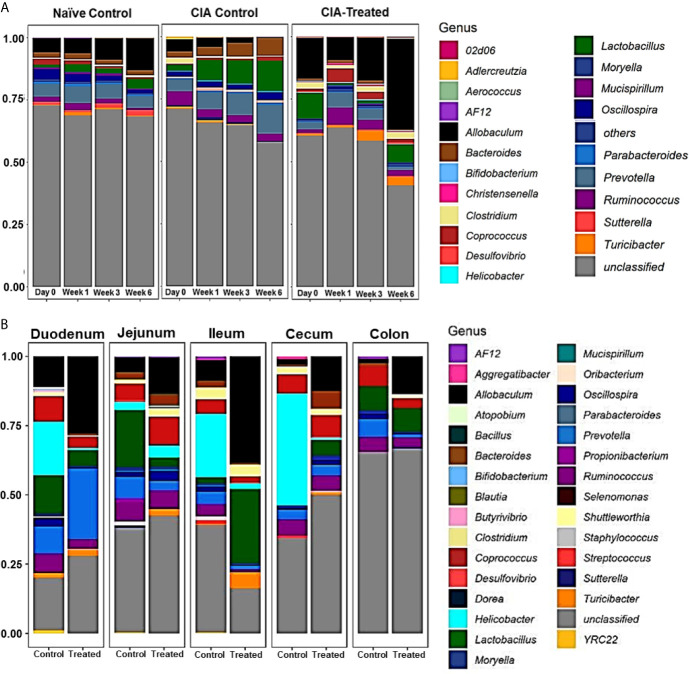
Relative abundance of microbial composition. **(A)** A comparison of the relative abundance of various genera and unclassified genus among naïve, arthritic non-treated [CIA control] and CII-immunized mice treated with MCI001 [CIA-treated] groups as various time points. N=5 for each group and time. **(B)** Relative abundance of microbial diversity of gut sections compared between arthritic non-treated and MCI001 treated mice. Y-axis represents the proportion of microbial composition.

Relative abundance analysis revealed that the dysbiosis due to CII-immunization was represented by an increase in *Prevotella* genus and a decrease in genus *Allobaculum*. DQ8 mice treated with MCI001, showed a reversal within 2 weeks of treatment with an increase in *Allobaculum* and a decrease in *Ruminococcus*, which resembles that of naïve mice. Interestingly, *P. histicola* treatment led to a decrease in the species belonging to genus *Prevotella* in fecal samples; this reduction was not observed in non-treated CIA mice (**Figures 3**, **4A**). Overall the observations depict a partial normalization of gut microbiota after treatment of CII-immunized DQ8 mice with *MCI001*. The genus *Allobaculum* presented the strongest correlation to healthy state as naïve and MCI001 treated mice showed similar abundance in comparison to arthritic mice (**Figure 3**). Variations in the relative abundances of significant genera at various time points in all the groups are illustrated in supplementary information (**Figures S1**–**S3**). Dysbiosis was also evident in phylum and family levels of relative abundance analysis (**Figure S4**). At the phylum level, MCI001 treated mice showed an increase in *Firmicutes*, predominantly represented by family *Erycepelotrichaceae and* the genus *Allobaculum*, after 6 weeks. Thus variation in the genus *Allobaculum* was directly proportional to the abundance of family *Erycepelotrichaceae*.

The present study is in confirmation with our previous observations exhibiting a protective impact of MCI001 treatment on arthritis (26). DQ8 mice treated with MCI001 did not show disease onset till 4 weeks post-immunization while the non-treated CII-immunized group developed arthritis after 2 weeks of immunization with all mice becoming arthritic by week 6 (**Figure S5**). In MCI001 treated group, delay in disease onset at week 3 correlated with a shift in fecal microbiome, where the restoration of eubiosis helped in delaying disease onset and reduced incidence. Treated mice showed 50% incidence by week 6 as compared to 100% arthritis in non-treated mice.

### *P. histicola* MCI001 Colonizes Duodenum and Enhances *Allobaculum* Abundance

**Figure 5:**
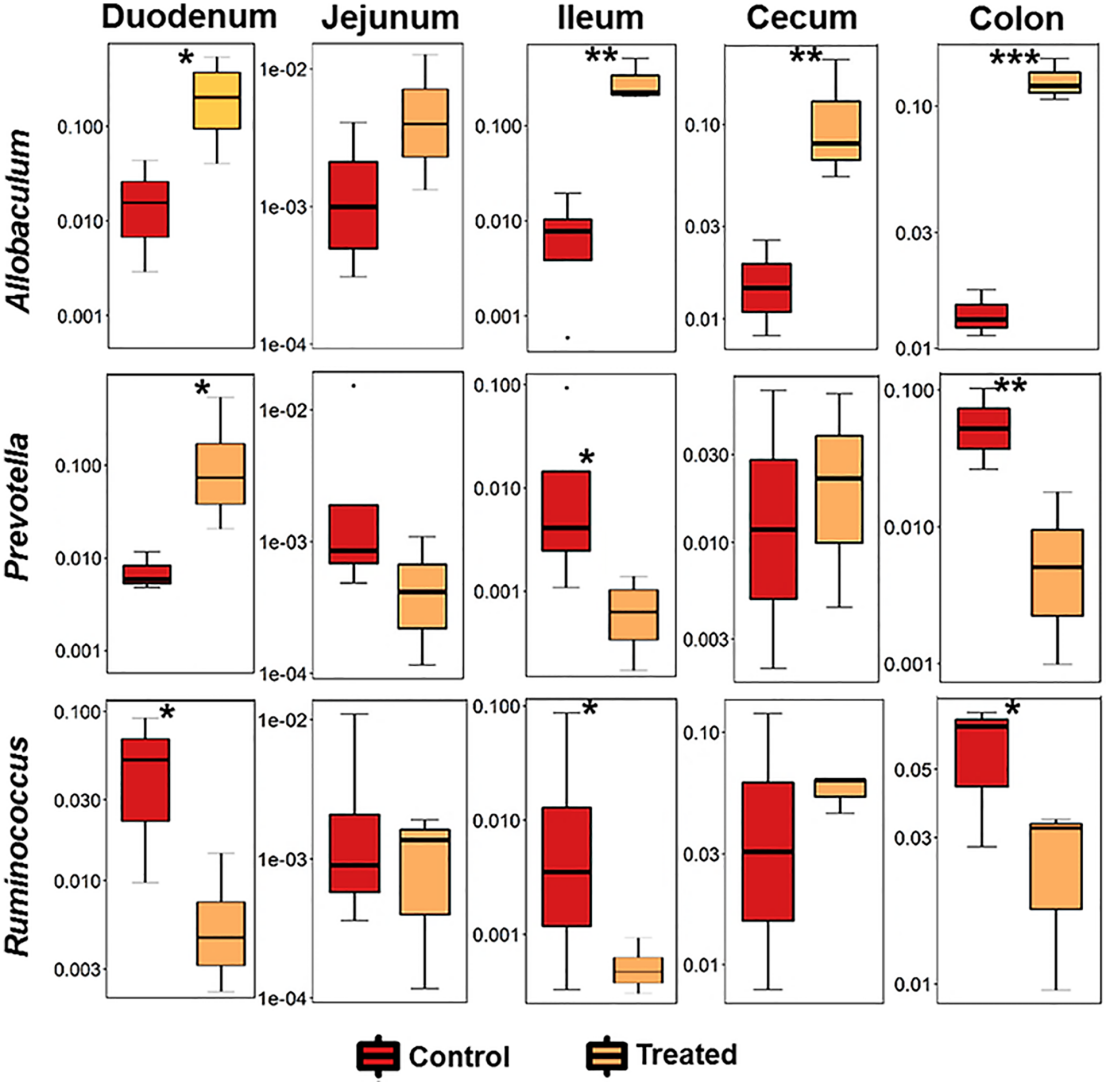
*P. histicola* colonizes the duodenum and expands *Allobaculum*. Relative abundance of significant bacteria compared between CIA control and MCI001 treated mice. N=3-4 for each group and time; statistical significance given as *P ≤ 0.05, **P ≤ 0.01 and ***P ≤ 0.001.

As MCI001 was isolated from a duodenal biopsy, we determined the site of colonization in DQ8 mice. Microbial composition of various gut sections was compared between non-treated arthritic and MCI001 treated DQ8 mice. MCI001 largely colonized in the duodenum of the small intestine and did not colonize the jejunum, ileum and cecum but showed some presence in the colon (**Figure 4B**). In duodenum and ileum, phyla *Bacteroidetes*, *Proteobacteria* and *Actinobacteria* were decreased in the MCI001 treatment group when compared to non-treated mice. However, the cecum showed an increase in *Bacteroidetes* and *Firmicutes* with a decrease in the abundance of *Proteobacteria* after MCI001 treatment (**Figure S6**). MCI001 treatment led to an expansion of *Allobaculum* in all gut sections of the treated group (**Figure 5**). Genus *Helicobacter* and *Lactobacillus* were decreased in the small intestine and cecum, while colon showed a decrease in *Desulfovibrio* and *Prevotella* genera in the treated group. As fecal samples mainly represent the colon microbiota, microbial diversity variations from the small intestine and cecum are not closely reflected in fecal samples. Our data suggest that analyzing both fecal and gut sections’ microbiota will help in interpreting gut modulatory effects precisely.

**Figure 6:**
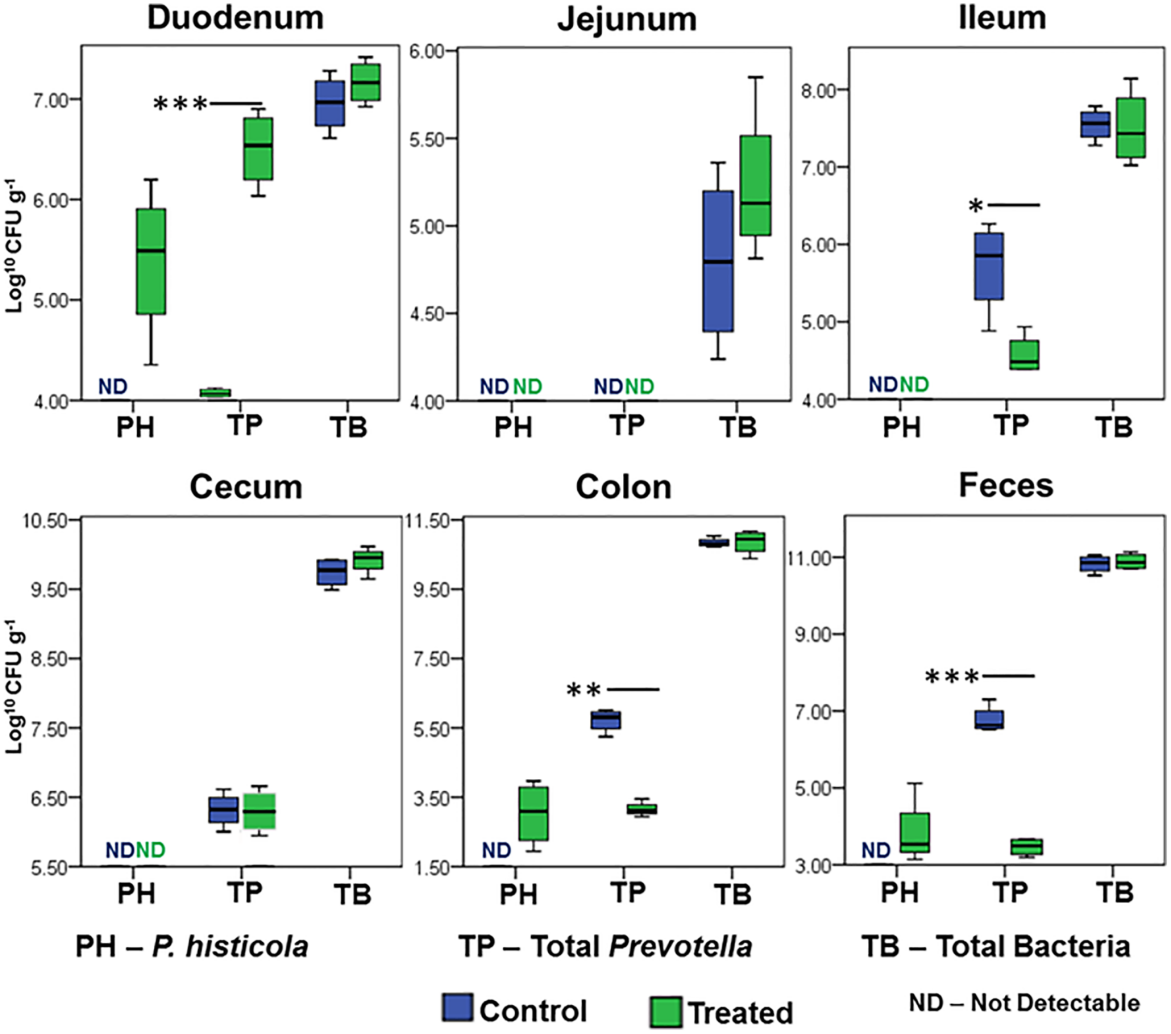
qPCR quantification of *P. histicola*, total *Prevotella*, and total bacterial load in gut sections. N=5 for each group and time; statistical significance given as *P ≤ 0.05, **P ≤ 0.01 and ***P ≤ 0.001.

To further define the niche for MCI001 colonization, qPCR analysis of gut sections was carried out (**Figure 6**). In qPCR analysis, *P. histicola* species was only observed in duodenum, colon and fecal samples of MCI001 treated mice while it was not detected in gut sections of non-treated and naïve mice. Interestingly, even though *P. histicola* colonized the upper and lower intestine, its colonization reduced the abundance of overall genus *Prevotella* in the ileum, colon, and feces. Jejunum showed lower bacterial population compared to other gut sections.

### *P. histicola* MCI001 Treatment Restores SCFA Levels

**Figure 7:**
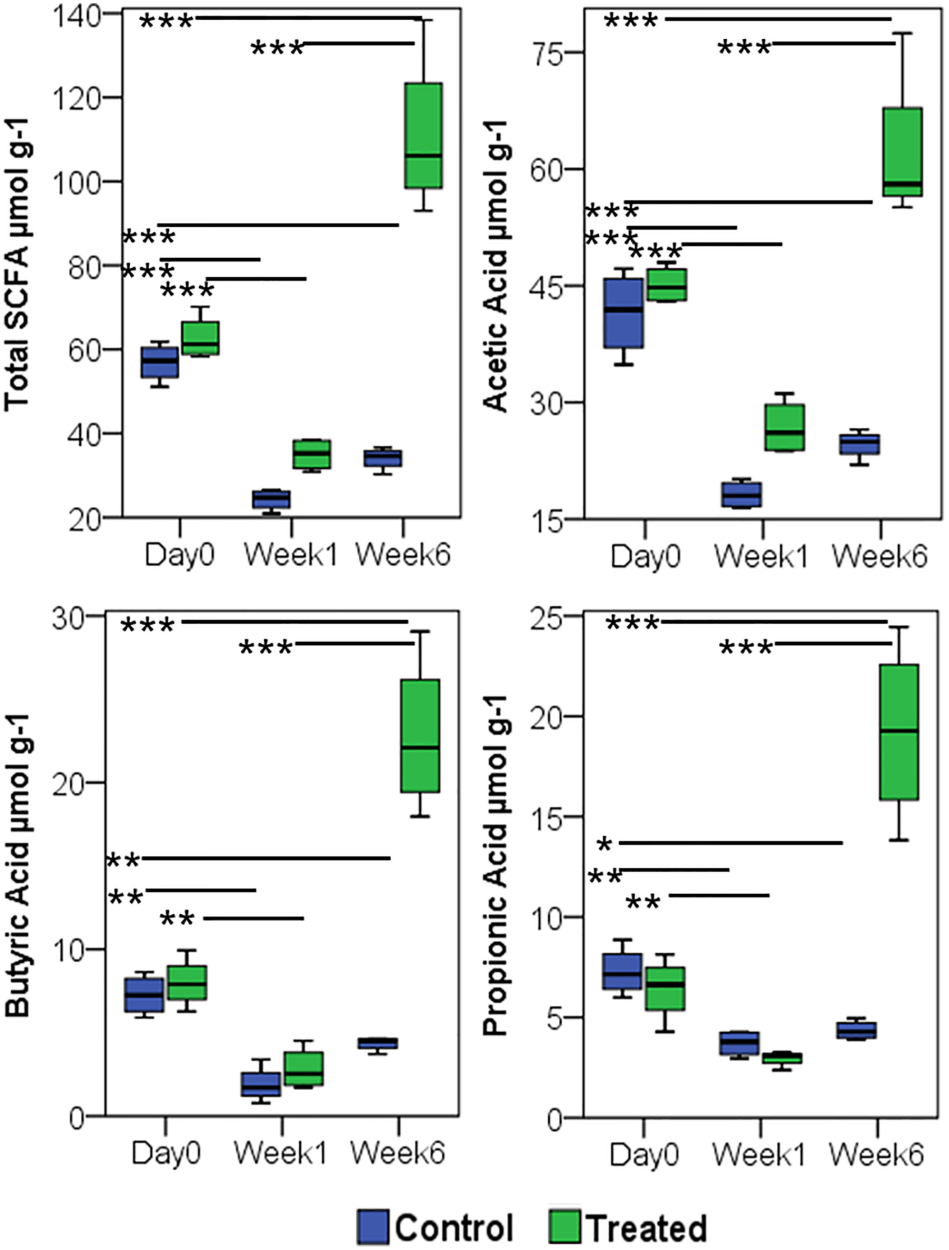
Treatment with *P. histicola* MCI 001 augments SCFA production. Quantification of SCFA from fecal samples, compared between arthritic (CIA control) and MCI001 treated group. N=5 for each group and time; statistical significance given as *P ≤ 0.05, **P ≤ 0.01 and ***P ≤ 0.001.

Fecal SCFA comparison between arthritic non-treated and MCI001 treated mice at various time points longitudinally from day 0 to week 6 showed a decrease in SCFA production in arthritic group after one week (**Figure 7**). A decrease in total SCFAs correlated with dysbiosis of the gut microbiome (**Figure 2**). However, after treatment with MCI001, SCFA levels showed an increased correlation with the restoration of eubiosis. A similar trend was observed with acetic acid, butyric acid, and propionic acid. Overall acetic acid production dominated followed by butyric acid and propionic acid. These changes can be directly correlated to MCI001 and the abundance of genus *Allobaculum* (**Figures 3**, **7**). Other SCFAs levels were low in concentration, below 4 µmol g-1 levels, and also did not show significant differences between selected time points.

## Discussion

Our previous study showed that MCI001 protects DQ8 mice from arthritis by generation of T regulatory cells in the periphery as well as synovium and reducing pro-inflammatory cytokines (26, 50). In the present study, we have shown that the P. histicola isolate MCI001 colonizes the same niche as in humans and delays the onset of CIA in DQ8 mice by modulating gut microbiome and SCFAs. Most studies focus on fecal microbiota which mostly represents the colon microbiome. However, fecal microbiome may not completely represent other parts of the gut, especially small intestine. Small intestine has low pH resulting in low abundance of commensals. Since small intestine has thinner mucus layer compared to the colon, it is much more sensitive to microbial interactions than large intestine and requires specific microbial populations for the maintenance of immune homeostasis (51–53). The changes in small intestinal physiology and microbiome can directly affect the colon microbiome. The MCI001 was isolated from the duodenum biopsy of a human and hence we posited that it might find the same niche in mice for colonization. The data presented here show that MCI001 explicitly colonizes duodenum of the treated DQ8 mice. Besides duodenum, MCI001 also colonized the colon and cecum, though it was not as dominant as in the duodenum. *P. histicola* dominated in the total bacterial composition of the duodenum even though genus *Prevotella* was decreased in the treated group suggesting *P. histicola* may compete with other species of *Prevotella* which may be pathogenic. Although the numbers of mice studied are small in numbers, this was supported by an increase in *Prevotella* in arthritic non-treated group indicating that native *Prevotella*, not belonging to *P. histicola*, in DQ8 mice may contribute to disease. One can envisage that the competitive reduction of other *Prevotella* members by MCI001 helps in reducing the disease severity. Indeed, our previous studies support this notion (26). However, another *Prevotella* species, *P. copri* has been implicated in RA pathogenesis (27, 54, 55). Taxonomically, one group of *Prevotella* [*P. melaninogenica* group] is commensal bacteria and another group of *Prevotella* [*P. intermedia* group] has been reported to have pathogenic characters (1). *P. histicola* belongs to *P. melaninogenica* group, phylogenetic and phenotypic analysis of the present study also support this statement (1).

The observations from this study indicate that MCI001 interactions restore gut microbial composition to that of naïve state and protect by interactions with other gut bacteria especially with the members of *Firmicutes* predominantly with genus *Allobaculum* which was increased in all parts of the gut except Jejunum. The data in the present study implicates a significant role of *Allobaculum*. While other genera showed variability and different trends at various time points, *Allobaculum* was increased in all gut sections in MCI001 treated group. A significant reduction in *Allobaculum* abundance after CII immunization suggests an evidence of dysbiosis as an expansion of *Allobaculum* in mice correlated with a delay in disease onset and lower disease severity and restoration of eubiosis in the treated group. In addition, changes in SCFAs levels were directly proportional to the alterations in *Allobaculum* abundance. These data point to *Allobaculum* as a key bacterium in the treated group and suggests that one of the mechanism by which MCI001 treatment protects from arthritis may be by influencing the growth of genus *Allobaculum*. However, we cannot rule out the role of other microbes in generating eubiosis.

Interaction between MCI001 and *Allobaculum* may be responsible for metabolic and consequently immune changes in the small intestine of the treated mice which drive the anti-inflammatory response. The observations in this study are reminiscent of the previous data in NOD mice where treatment of obese mice with high fiber diet led to weight loss which was associated with the colonization of *Prevotella* and *Allobaculum* (56, 57). *Allobaculum* is one of the high butyrate producing *Firmicute* and is an active glucose assimilator in the gut (58–60). The present study also affirmed that the increase in *Allobaculum* correlated with an increase in SCFA production, especially butyrate. *Allobaculum* is a mouse commensal and has properties similar to *Faecalibacterium*, the most abundant commensal in the healthy human gut (61). Both *Faecalibacterium* and *Allobaculum* are *Firmicutes* that are butyrate producers and share metabolic similarities in the gut. Both of these bacteria can coexist and dominate in the guts of mice treated for metabolic disorders (57).

One can speculate that an essential metabolite produced by *P. histicola* leads to the expansion of *Allobaculum* in the host’s gut augmenting butyrate production as shown in MCI001 treated mice which leads to generation of T regulatory cells as shown previously (**Figure S7**) (26). Butyrate is widely reported SCFA for its interaction with the gut immune system in maintaining immune homeostasis (19, 20, 62, 63) and is a significant energy source for many commensal bacteria in the gut (63). Duodenum colonized *P. histicola* may be involved in the breakdown of undigested complex carbohydrates and oligosaccharides from the stomach, a common characteristic of a commensal present in the small intestine (64), and thus may aid in carbohydrate digestion leading to an increase in glucose and other simple carbohydrates. Biochemical analysis supports that MCI001 produces a wide variety of enzymes that are involved in the degradation of complex carbohydrates. An increase in glucose levels could support the growth of native *Allobaculum* which produces butyrate, thereby increasing the growth of natural commensals to restore commensal bacterial composition. As *Allobaculum* is an extraordinary glucose assimilator, it may decrease the availability of glucose to CD4 T cells for proliferation and cytokine production in RA (65). Our previous study supports this observation as treatment with MCI001 downregulated Th17 and increased T regulatory cells (8, 26).

Based on the above observations, we explored if MCI001 is a bio-therapeutic agent. Biochemical characters of the strain MCI001 prove its versatility in growing at various pH and metabolic conditions supported by colonization in duodenum in treated mice. Besides the colonization characteristics, MCI001 can support the health of the host by other functions such as glycerol metabolism, producing vitamins and SCFA, degrading mucin and interacting with other commensal bacteria. Glycerol utilizing character of MCI001 increases its acetate productivity which may be utilized by beneficial *Firmicutes* commensals such as *Faecalibacterium* and *Allobaculum.* Acetate is the major SCFA [over 50%] and represents a healthy gut (17, 60, 66). Furthermore, excess acetate can be converted to butyrate by the microbial enzymes in the gut (17, 60). Thus glycerol metabolism and acetate production characters of MCI001 represent beneficial actions in maintaining gut homeostasis.

Biotin and Folate production by MCI001 increases its feasibility of therapeutic applications. While many bacteria in the colon can produce vitamins, its absorption is very low in the colon. Most of the essential vitamins are absorbed in the proximal small intestine (67–69),. Also, Folate is an essential vitamin B required for many metabolic reactions such as nucleic acid synthesis, amino acid synthesis and protecting the genome from free radicals (70, 71). Patients with RA being treated with Methotrexate [MTX] are given folate supplements to avoid folate deficiency and MTX-dependent reduction in the production of dihydrofolate reductase and other folate-dependent enzymes (72, 73). As the microbial production of folate is controlled by environmental needs; it is considered to be a critical character for a probiotic bacteria (68). Taking into consideration the efficiency of MCI001 in colonizing duodenum; MCI001 can be used as a vital alternative in replacing chemical folate supplementation in RA patients on MTX treatment. In *in-vitro* studies, MCI001 did not show any degradation activity for MTX and MTX did not show any antimicrobial properties on MCI001 [Data not shown].

A human gut-derived anaerobic commensal for the therapeutic purpose is a novel concept. Though upper gut has lower microbial load and diversity, it is immunologically active and can have a significant impact on the immune function. Based on the discussed characters of MCI001 and its ability to modulate immune system and treat autoimmune diseases in mouse models, *P. histicola* MCI001, a microbe in the upper gut, could be as a promising bio-therapeutic agent for humans.

## Conclusion

Our data demonstrate that treatment with *P. histicola* MCI001 partially normalizes the microbiota of arthritic gut to naïve state in DQ8 mice. A similar restoration has been demonstrated in RA patients treated with Methotrexate (11, 74). Microbial restoration after treatment with P. histicola also enhanced the production of SCFAs. The *P. histicola* isolate reported here colonized the duodenum, a site from where it was isolated from human biopsy, suggesting that the immune response modulation *via* the upper gut may be a viable therapeutic option for RA.

## Data Availability Statement

The original contributions presented in the study are publicly available. This data can be found here: NCBI BioProject accession PRJNA691623 (https://www.ncbi.nlm.nih.gov/bioproject/PRJNA691623).

## Ethics Statement

The animal study was reviewed and approved by IACUC, Mayo Clinic.

## Author Contributions

VT, JM and BB designed the concept. BB and VT designed the research experiments. BB, EM, and DL carried out most of the experimental work. BB, RB, JC, PJ, and VT analyzed the data. BB and VT wrote the manuscript with the input of co-authors. All authors contributed to the article and approved the submitted version.

## Funding

The work was supported by funds from the Department of Defense grants W81XWH-10-1-0257 and W81XWH-15-1-0213 to VT.

## Conflict of Interest

Authors VT, JM and EM associated with this project and Mayo Clinic has a Financial Conflict of Interest in Prevotella histicola used in the research and that the investigator(s) and Mayo Clinic may stand to gain financially from the successful outcome of the research.
