# Supplementary material for: Prevotella histicola Protects From Arthritis by Expansion of Allobaculum and Augmenting Butyrate Production in Humanized Mice
Source: Front Immunol. 2021 May 4;12:609644. doi: 10.3389/fimmu.2021.609644 (PMC8130672; doi:10.3389/fimmu.2021.609644)
Supplement: Supplementary file 1 [file DataSheet_1.doc]

**Supplementary Material**

***Prevotella histicola* MCI 001 protects from arthritis by expansion of *Allobaculum* and modulation of metabolic profile**

Baskar Balakrishnan^1^, David Luckey^1^, Rahul Bodhke^1,2^, Jun Chen^3^, Eric Marietta^4^, Patricio Jeraldo^5^, Joseph Murray^4.^, Veena Taneja^1*^

^1^Department of Immunology, Mayo Clinic, Rochester, MN, USA

^2^National Center for Microbial Resource, National Center for Cell Science, Pune, India

^3^Department of Biomedical Statistics and Informatics, Mayo Clinic, Rochester, MN, USA

^4^Department of Medicine, Division of Gastroenterology and Hepatology, Mayo Clinic, Rochester, MN, USA

^5^Department of Surgery, Division of surgical research, Mayo Clinic College of Medicine, Rochester, MN, USA

***Corresponding Author Address**

Veena Taneja

Department of Immunology

Mayo Clinic

200 First St. SW

Rochester, MN55905.

Tel 507 284 4541

Email [taneja.veena@mayo.edu](mailto:taneja.veena@mayo.edu)

**Supplementary Table S1.** Biochemical characteristics of *P. histicola* MCI 001, in production of enzymes and carbohydrate utilization. Listed the results of different API kits used in the study.

**Table S1A. API 20 A assay of P. histicola MCI 001 showed profile ID 46124200.**

| **Test** | **Result** |
| --- | --- |
| IND- INDole | - |
| URE- UREa | - |
| GLU- D-Glucose | + |
| MAN- D-MANnitol | - |
| LAC- D-LACtose | + |
| SAC- D-SACcharose | + |
| MAL- D-MALtose | + |
| SAL- SALicin | - |
| XYL- D-Xylose | - |
| ARA- L-ARAbinose | - |
| GEL- GELatin | + |
| ESC- Asculin | **-** |
| GLY- GLYcerol | w+ |
| CEL- D-Cellobiose | - |
| MNE- D-MaNnosE | + |
| MLZ- D-MeLeZitose | - |
| RAF- D-Raffinose | + |
| SOR- D-SORbitol | - |
| RHA- L-RHAmnose | - |
| TRE- D-TREhalose | - |
| CAT- CATalase | - |
| SPOR- SPORe formation | - |
| GRAM- GRAM Reaction | - |
| COCC- Morphology | - |

**Table S1B.** Rapid ID 32 A assay of *P. histicola* MCI 001 showed profile ID 4707 4502 22.

| **Test** | **Result** |
| --- | --- |
| URE- UREase | - |
| ADH- Arginine DiHydrolase | - |
| α-GAL- α-GALactosidase | + |
| β-GAL- β-GALactosidase | + |
| ßGP- ß-Galactosidase 6 Phosphate | + |
| αGLU- α-GLUcosidase | + |
| βGLU- β-GLUcosidase | - |
| αARA- α-ARAbinosidase | - |
| βGUR- β-GlucURonidase | - |
| ßNAG- N-Acetyl-ß-Glucosaminidase | + |
| MNE- D-mannose MaNnosE fermentation | + |
| RAF- D-raffinose RAFfinose fermentation | + |
| NIT- Reduction of NITrates | - |
| IND- INDole production | - |
| PAL- ALkaline Phosphatase | + |
| ArgA- Arginine Arylamidase | + |
| ProA Proline Arylamidase | - |
| LGA- Leucyl Glycine Arylamidase | + |
| PheA- Phenylalanine Arylamidase | - |
| LeuA- Leucine Arylamidase | - |
| PyrA- Pyroglutamic acid Arylamidase | - |
| Try- Tyrosine Arylamidase | - |
| AlaA- Alanine Arylamidase | + |
| GlyA- Glycine Arylamidase | - |
| GDC- Glutamic acid DeCarboxylase | - |
| αFUC- α-FUCosidase | + |
| HisA- Histidine Arylamidase | - |
| GGA- Glutamyl Glutamic acid Arylamidase | + |
| SerA- Serine Arylamidase | - |

**Table S1C. API ZYM assay of *P. histicola* MCI 001.**

| **Test** | **Result** |
| --- | --- |
| Alkaline phosphatase | + |
| Esterase (C 4) | + |
| Esterase Lipase (C 8) | - |
| Lipase (C 14) | - |
| Leucine arylamidase | + |
| Valine arylamidase | - |
| Cystine arylamidase | - |
| Trypsin | - |
| α-chymotrypsin | - |
| Acid phosphatase | + |
| Naphthol-AS-BI-phosphohydrolase | + |
| α-galactosidase | + |
| ß-galactosidase | + |
| ß-glucuronidase | - |
| α-glucosidase | - |
| ß-glucosidase | - |
| N-acetyl-ß-glucosaminidase | + |
| α-mannosidase | - |
| α-fucosidase | - |

**Table S1D.** Carbohydrate utilization study of *P. histicola* MCI 001.

| **Test** | **Result** |
| --- | --- |
| GLY GLYcerol | w+ |
| ERY ERYthritol | - |
| DARA D-ARAbinose | - |
| LARA L-ARAbinose | - |
| RIB D-RIBose | - |
| DXYL D-XYLose | - |
| LXYL L-XYLose | - |
| ADO D-ADOnitol | - |
| MDX Methyl-βD-Xylopyranoside | - |
| GAL D-GALactose | + |
| GLU D-GLUcose | + |
| FRU D-FRUctose | + |
| MNE D-MaNnosE | + |
| SBE L-SorBosE | - |
| RHA L-RHAmnose | - |
| DUL DULcitol | - |
| INO INOsitol | - |
| MAN D-MANnitol | - |
| SOR D-SORbitol | - |
| MDM Methyl-αD-Mannopyranoside | - |
| MDG Methyl-αD-Glucosamine | - |
| NAG N-AcetylGlucosamine | + |
| AMY AMYgdaline | - |
| ARB ARButine | - |
| ESC ESCuline | - |
| SAL SALicine | - |
| CEL D-CELobiose | - |
| MAL D-MALtose | + |
| LAC D-LACtose | + |
| MEL D-MELibiose | - |
| SAC D-SACcharose | + |
| TRE D-TREhalose | - |
| INU INUline | + |
| MLZ D-MeLeZitose | - |
| RAF D-RAFfinose | + |
| AMD AMiDon | + |
| GLYG GLYcoGene | + |
| XLT XyLiTol | - |
| GEN GENtiobiose | - |
| TUR D-TURanose | + |
| LYX D-LYXose | - |
| TAG D-TAGatose | - |
| DFUC D-FUCose | - |
| LFUC L-FUCose | - |
| DARL D-ARabitol | - |
| LARL L-ARabitol | - |
| GNT potassium GlucoNaTe | - |
| 2KG potassium 2-KetoGluconate | + |
| 5KG potassium 5-KetoGluconate | - |


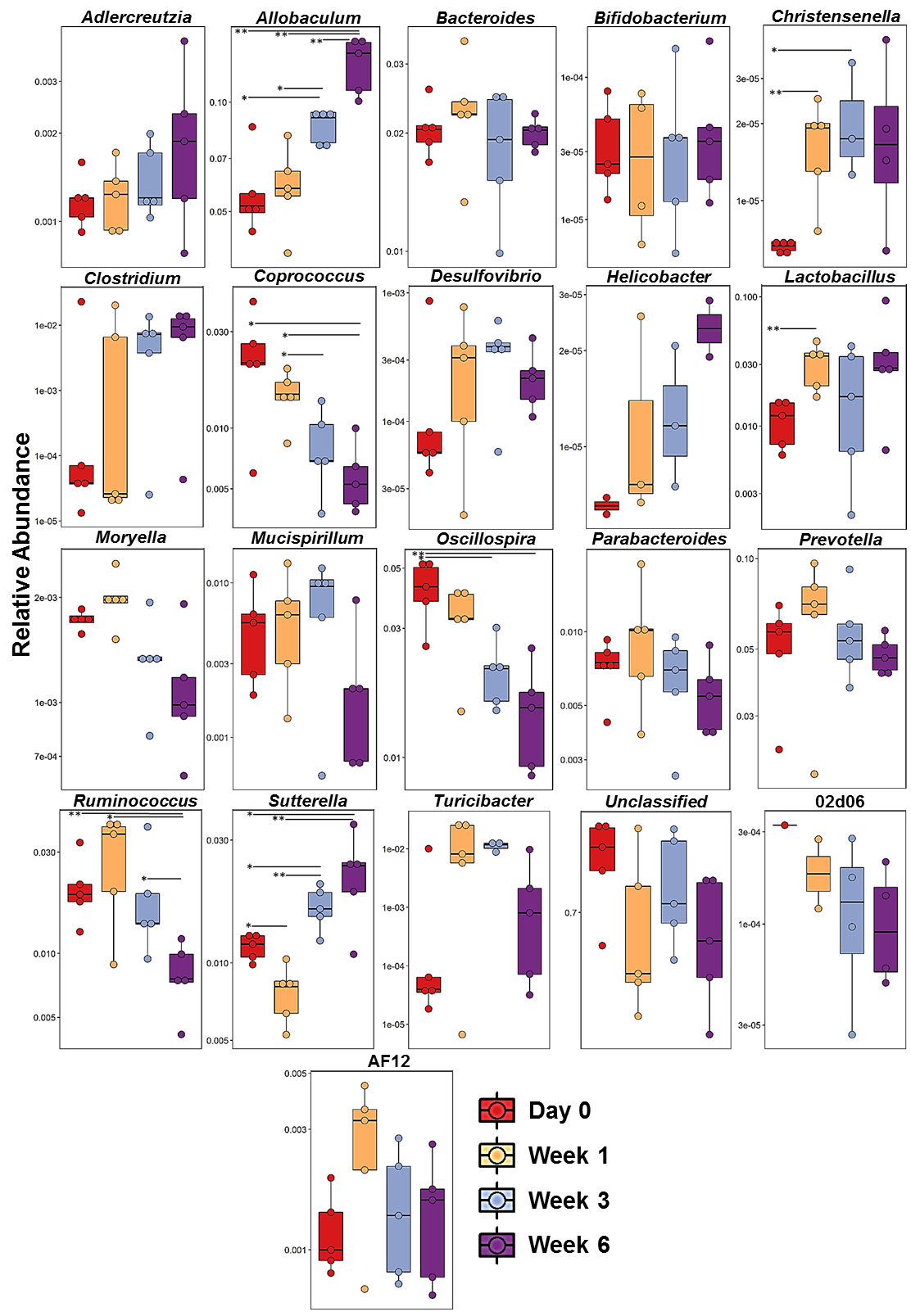


**Supplementary Figure S1** Relative abundance of dominant genera in fecal samples of Naïve control group. Fecal samples collected over a period of 6 weeks at varying times were used for 16S sequencing and relative abundance of the compared between time points of the longitudinal study. There was a gradual increase in the abundance of the genus *Allobaculum* and *Sutterella*, with a decrease in *Coprococcus* and *Ruminococus* over the duration of the follow up of DQ8 mouse representing healthy microbial changes in the gut.


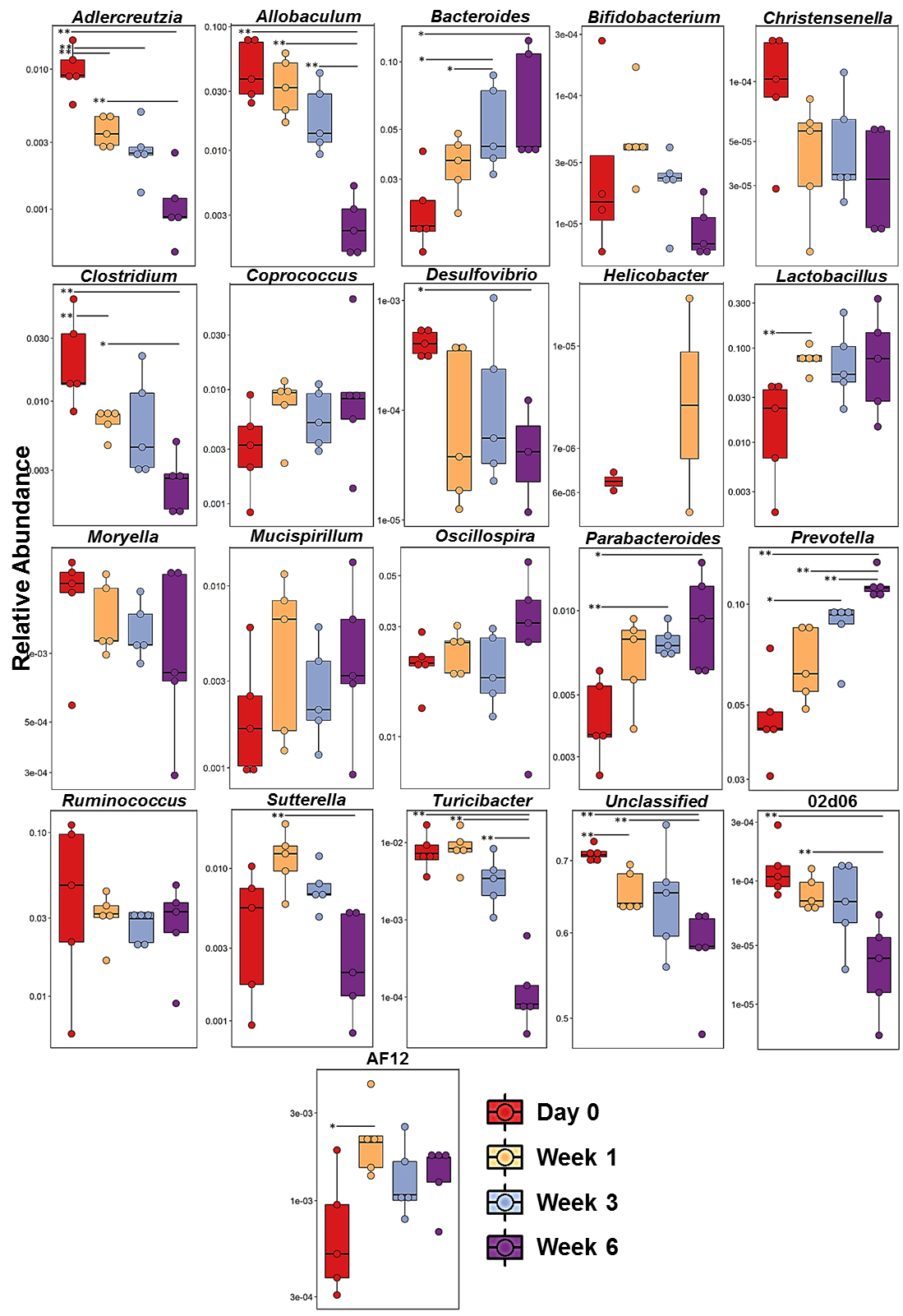


**Supplementary Figure S2** Relative abundance of the dominant genus in fecal samples of the CIA control group in the longitudinal study. Fecal samples collected over a period of 6 weeks were used for 16S sequencing and relative abundance of the dominant genera in the CIA control group was compared over time. The data suggest that induction of arthritis led to a decrease in the abundance of *Adlercreutzia*, *Allobaculum*, *Clostridium,* and *Turicibacter* with an increase in *Bacteroides*, *Parabacteroides,* and *Prevotella,* thu*s* representing dysbiosis in diseased condition.


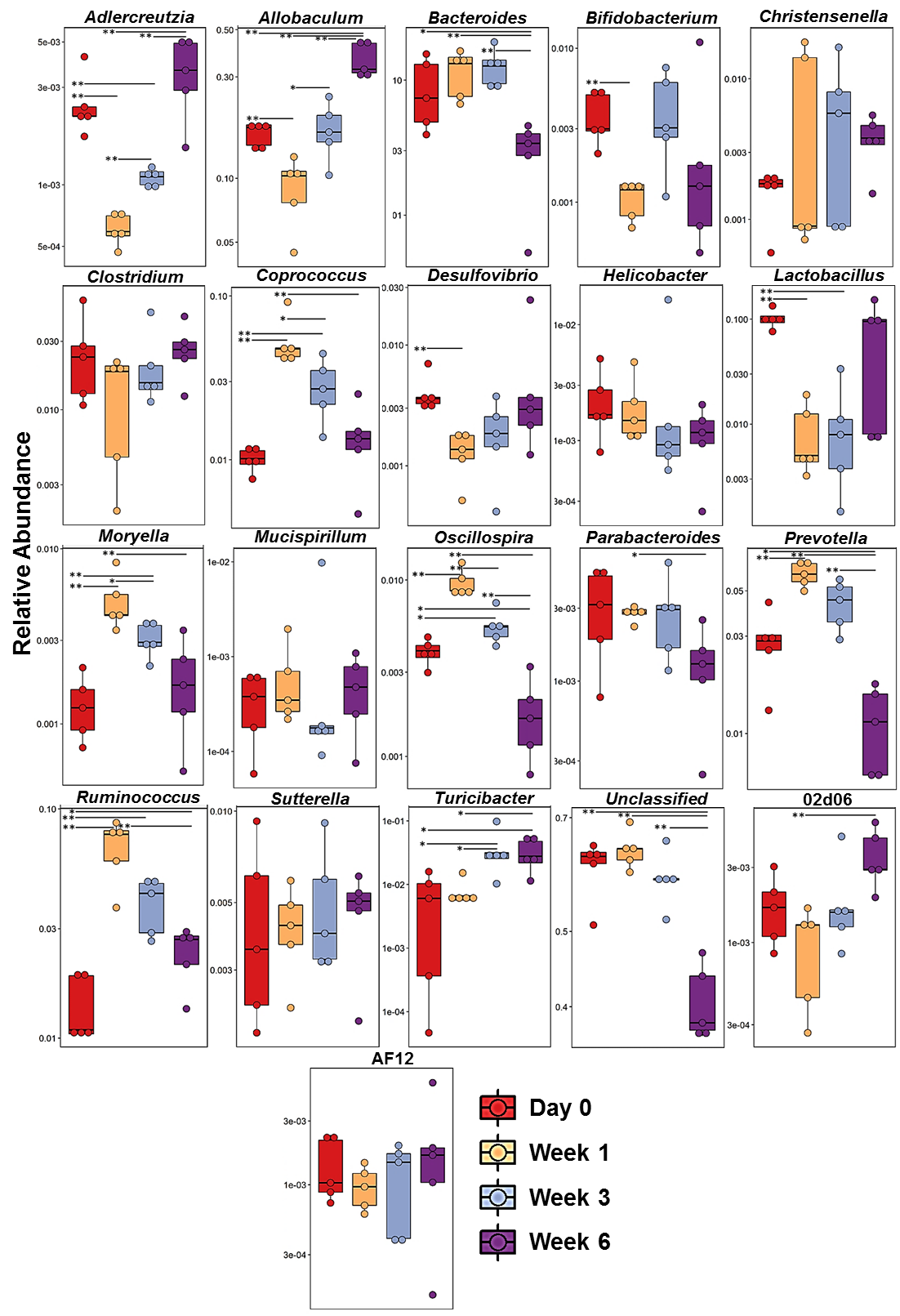


**Supplementary Figure S3** The relative abundance of the dominant genus in fecal samples of the MCI 001 treated group. Fecal samples were collected from naïve DQ8 mice, day 0, and then mice were immunized with type II collagen to induce arthritis. Immunization with collagen caused dysbiosis after a week by as shown by the decrease in the genus *Adlercreutzia* and *Allobaculum and an* increase in *Coprococcus* and *Ruminococcus. Treatment with P. histicola* MCI 001 started from week 2 and continued to week 6. Fecal samples collected (week 3 and week6) after treatment with MCI001 partially restored eubiosis by increasing the abundance of *Adlercreutzia* and *Allobaculum* and decreasing *Coprococcus* and *Ruminococcus*


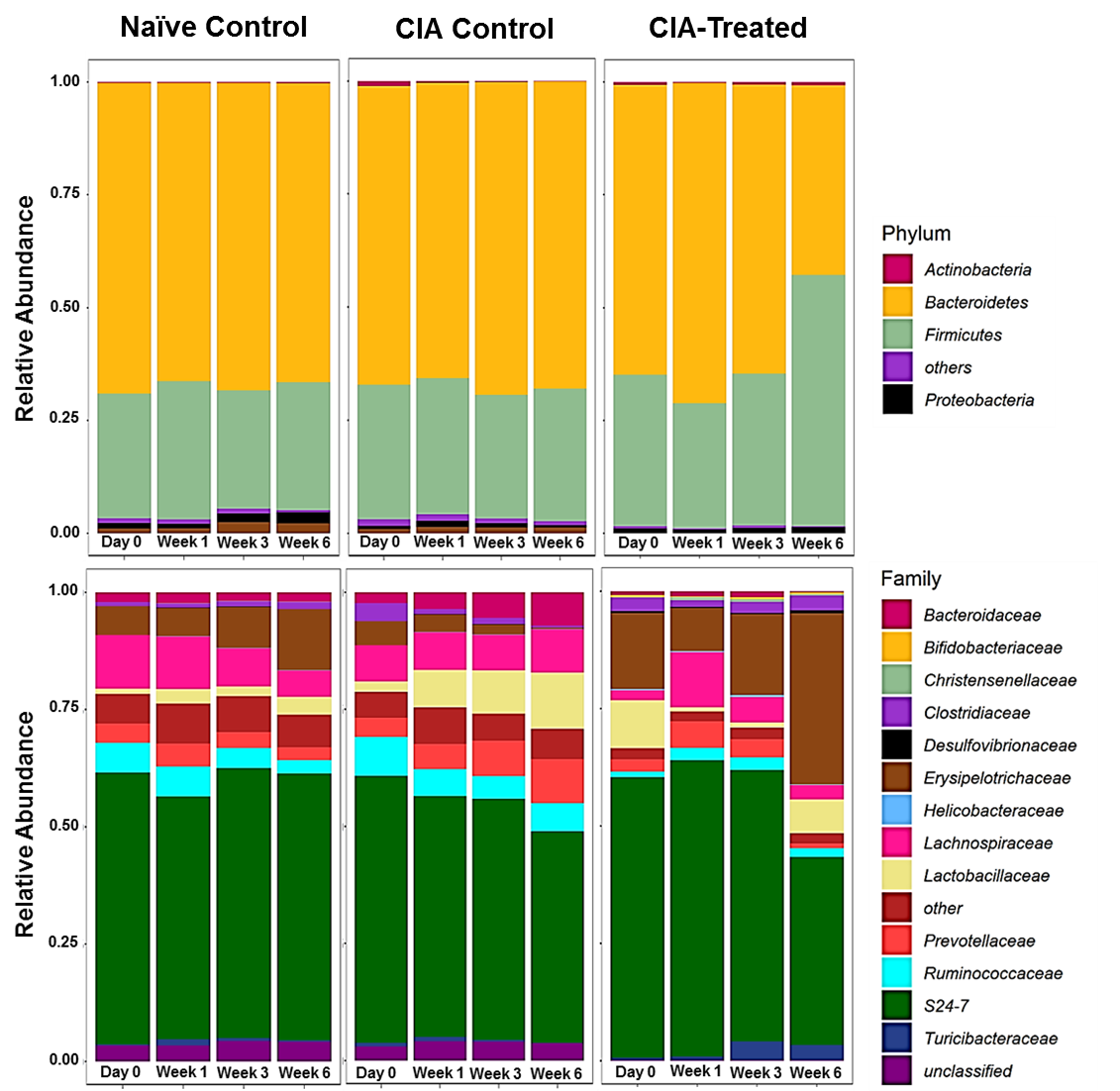


**Supplementary Figure S4** Relative abundance of phylum and family level diversity in fecal samples collected longitudinally over a period of 6 weeks. Naive DQ8 mice did not show any significant changes in phylum-level diversity over a period of 6 weeks. At the family level, *Erysipelotrichaceae* increased over the age of naïve mice which is supported by the abundance of genus *Allobaculum*. On the other hand, in arthritic non-treated mice, the microbial profile showed a reduced abundance of the *Erysipelotrichaceae* over the 6 weeks period, representing dysbiosis. Arthritis-induced DQ8 mice treated with MCI 001 restored the abundance of family *Erysipelotrichaceae* within 2 weeks of treatment (week 3) representing microbial restoration. The abundance of *Erysipelotrichaceae* increased after five weeks of *P. histicola MCI001* treatment (week 6) representing the trend of Naïve mice eubiotic condition.


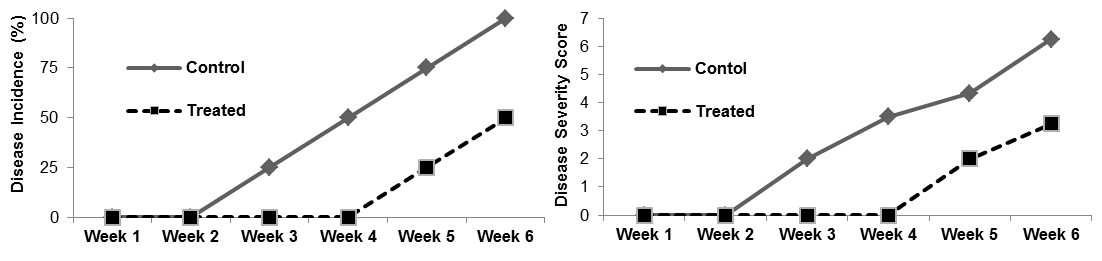


**Supplementary Figure S5** Arthritis onset and disease severity in *P. histicola* MCI001 treated and non-treated (control) arthritis-induced DQ8 mice. CIA control mice exhibited the onset of disease from week 3. Whereas *P. histicola* MCI 001 treatment delayed the onset of disease and developed the milder disease.


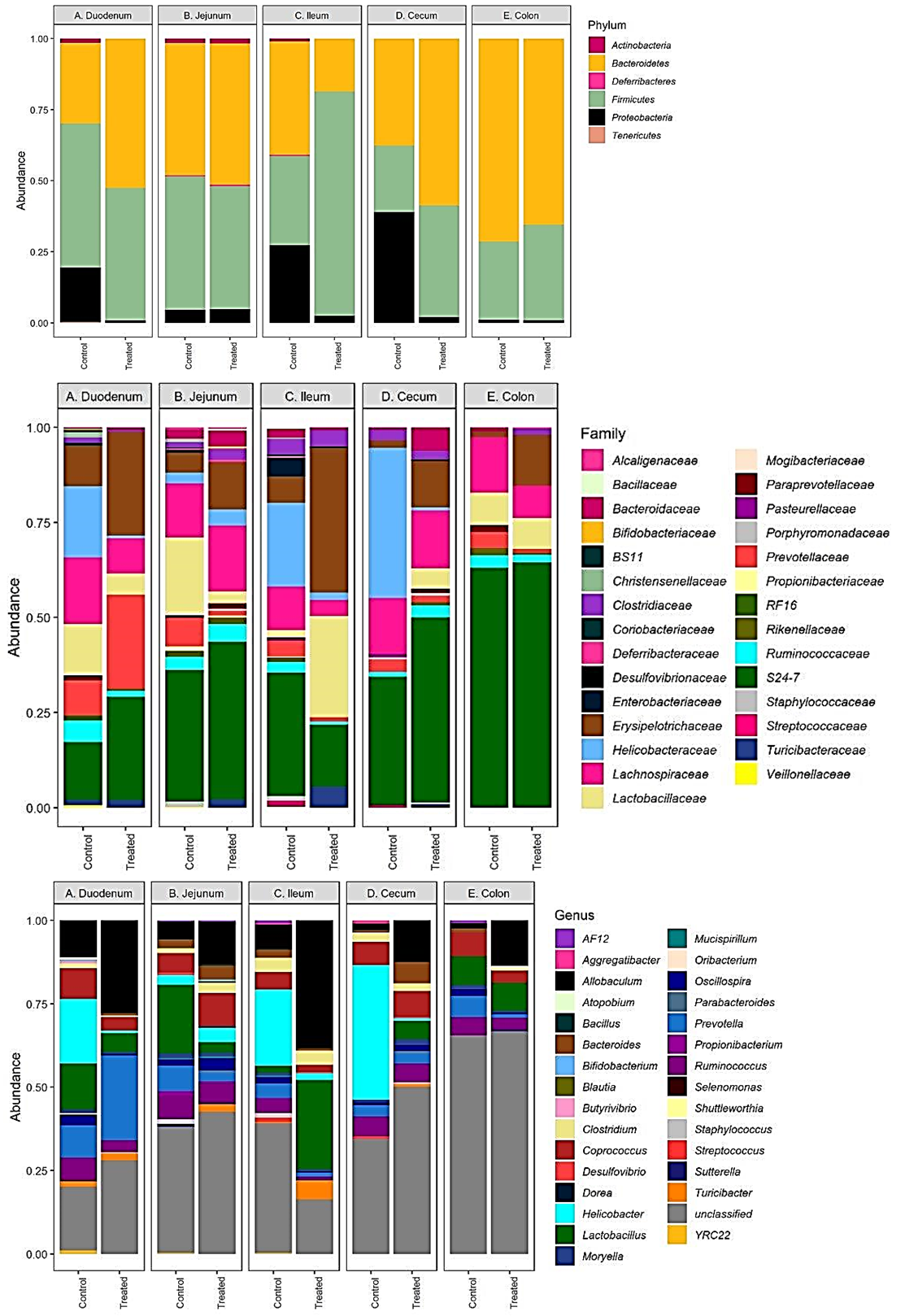


**Supplementary Figure S6** Relative abundance of phylum and family level diversity of gut sections (duodenum, Jejunum, Ileum, Cecum and colon) microbiome analysis. At the termination of the experiment, DQ8 mice in the arthritic non-treated (control) and MCI001 treated (Treated) groups were used for 16S sequencing of the microbial profile of the various gut sections to define changes incurred due to treatment. All gut sections showed the difference between control and treated groups in both phylum and family level diversity. Family *Erysipelotrichaceae was* increased in all the gut sections, similar to the observations for fecal samples. At the phylum level, *Proteobacteria was* increased in control mice representing diseased condition and dysbiosis. After treatment with MCI 001, gut sections showed a decrease in *Proteobacteria* abundance signifying the gut modulatory effect of *P. histicola* MCI 001 in restoring eubiosis in CIA with implications for treatment of RA.


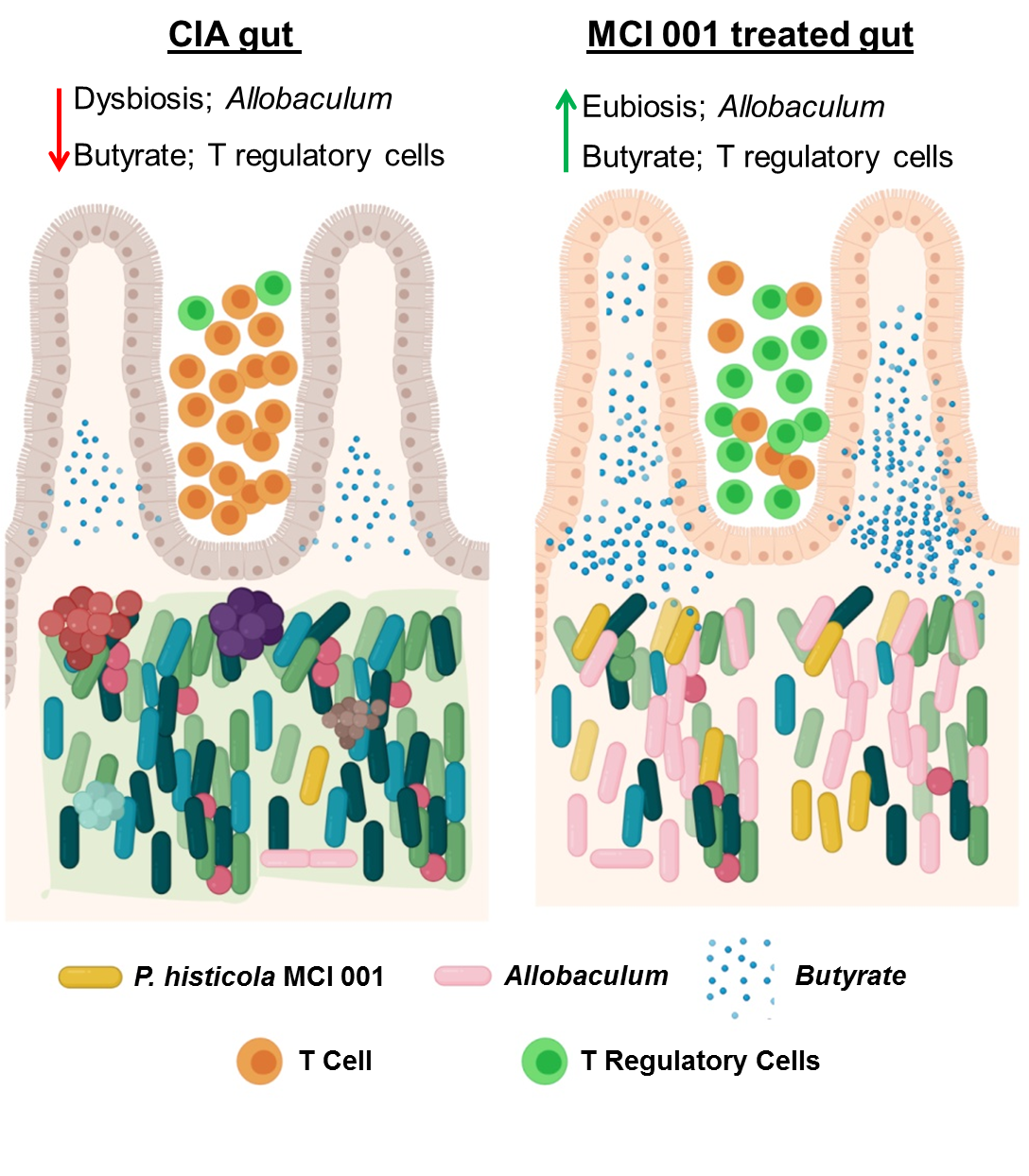


**Supplementary Figure S7** Mechanism of action involved MCI 001 treatment process. The CIA gut represents dysbiosis by showing a lower abundance of genus *Allobaculum* and reducing gut butyrate leading to lesser regulatory T cells in the gut. This microbial, metabolic, and immune disparity increases disease severity. In comparison, MCI 001 treated gut showing the partial restoration of the gut microbiome by increased abundance of *Allobaculum*. *Allobaculum* increases butyrate production in the gut. Increased butyrate, in turn, helps in increasing regulatory T cells in the gut and showing reducing disease severity than the CIA group.
